# Using molecular methods to diagnose, classify, and treat neonatal sepsis: a scoping review

**DOI:** 10.3389/fped.2025.1625449

**Published:** 2025-08-25

**Authors:** Catherine Y. Lee, Sidney E. Zven, Shreyas A. Sathya, Danielle Abukhalaf, Sneha Sahoo, Pratyusha Samal, Stephanie M. Prescott

**Affiliations:** ^1^ Morsani College of Medicine, University of South Florida, Tampa, FL, United States; ^2^ Department of Pediatrics, Walter Reed National Military Medical Center, Specialized Services University School of Medicine, Bethesda, MD, United States; ^3^ College of Arts and Sciences, University of South Florida, Tampa, FL, United States; ^4^ Department of Nursing, University of South Florida College of Nursing, Tampa, FL, United States; ^5^ Department of Neonatal Research, Inova Health Services, Falls Church, VA, United States

**Keywords:** genomics, metabolomics, transcriptomics, microbiome, multiomic, neonatal sepsis, neonates

## Abstract

**Introduction:**

Neonatal sepsis is a dysregulated immune response to bloodstream infection causing serious disease and death. Our review seeks to integrate the knowledge gained from studies of multiple molecular methods- such as genomics, metabolomics, transcriptomics, and the gut microbiome- in the setting of neonatal sepsis that may improve the diagnosis, classification, and treatment of the disease. Sepsis claims over 200,000 lives annually worldwide and remains a top 10 cause of infant mortality in the US. Diagnosis and treatment of neonatal sepsis remains a challenge as its mechanisms are poorly understood.

**Methods:**

We conducted a scoping review of literature published between 2018 and 2024. Of 1,043 articles screened, 30 were included in the final review.

**Results:**

The gut microbiome is associated with both pathogenicity and protection in the setting of neonatal sepsis, while expression levels of immune response and regulation help classify neonatal response to septic events. Metabolomic studies reveal possible biomarkers to detect, classify, and predict neonatal sepsis morbidity and mortality, and proteomic studies confirm mechanisms predicted by the other models.

**Discussion:**

Studies using molecular methods foster greater understanding of neonatal sepsis and show promise to improve diagnosis, classification, and therapeutic intervention. Future research using multi-omic analyses may further elucidate the development and progression of inflammatory processes that occur as sepsis progresses.

## 1 Introduction

Neonatal sepsis is a leading cause of mortality among infants worldwide, accounting for roughly 227,000 deaths in 2019, of which only 740 deaths occurred in North America (1). A 2021 meta-analysis estimates a mortality rate of 17.6% with deaths disproportionately affecting middle and low-income countries (2). In the US, the CDC reports neonatal sepsis as a top 10 cause of infant mortality as of 2022 (3). Neonatal sepsis is classified as early-onset (EOS) or late-onset sepsis (LOS); EOS occurs within the first 72 h of life, typically resulting from exposure to the maternal vaginal tract during delivery. In contrast, LOS arises after 72 h and is generally acquired postnatally through environmental exposure, particularly through nosocomial transmission in neonatal intensive care units (NICUs).

The diagnosis, treatment, and prevention of neonatal sepsis continues to pose significant challenges. Although a positive blood culture remains the gold standard for diagnosis, its sensitivity is limited by factors such as inadequate blood volume (4), low bacterial load, and the fastidious nature of certain pathogens (5). As a result, false negative cultures are common (5) and low colony count bacteremia represents the majority of neonatal sepsis cases (6), remaining undetected in up to 60% of cases (7). In these patients, clinicians must make the diagnosis of sepsis on clinical signs and non-specific laboratory markers, most commonly neutrophil counts and acute phase reactants (8). Despite a 2016 international consensus redefining sepsis and septic shock as a “life-threatening organ dysfunction caused by a dysregulated host response to infection” (9), blood culture positivity remains central to both diagnostic criteria and therapeutic decision making. In fact, a review of 80 randomized control trials identifies positive blood culture as the most frequently used criterion for defining neonatal sepsis, followed by clinical and laboratory indicators (8).

Neonates primarily depend on an immature innate immune system for defense against pathogens, as their adaptive immunity is underdeveloped. Their reliance on innate immunity contributes to their heightened vulnerability to sepsis and the associated increased mortality among this patient population (10, 11). Several acute phase reactants such as C-reactive protein and procalcitonin have been studied for their diagnostic utility as biomarkers of sepsis (12). However, tests to determine levels of such biomarkers in blood often require serial measurements, as acute phase reactants increase in response to early inflammatory signals occurs gradually (12). Moreover, these biomarkers often lack specificity and may be elevated in response to non-infectious factors such as perinatal stress, delivery trauma, or neonatal surgery, limiting their diagnostic utility (12). Empiric antibiotic therapy remains the standard initial treatment when clinical signs of sepsis are present (13). For EOS, regimens typically include broad-spectrum antibiotics such as ampicillin and gentamicin, with a third-generation cephalosporin added if meningitis is suspected (13). In cases of LOS, similar broad coverage of gram-positive, gram-negative, and anaerobic organisms is generally initiated (13). Preventative strategies for EOS frequently involve maternal intrapartum antibiotic prophylaxis and, in some cases, neonatal antibiotics (13, 14). However, the widespread use of broad-spectrum antibiotics has contributed to the emergence of drug resistant pathogens in neonatal populations (15, 16). Furthermore, early life exposure to antibiotics, particularly within the first two years of life, has been associated with increased risks of long term adverse outcomes, including asthma, allergic disease, and obesity (17–19).

The need for prudent antibiotic stewardship, coupled with the inherent limitations of current diagnostic modalities in neonatal sepsis underscores the imperative to develop more precise diagnostic methodologies, targeted therapeutic strategies, and effective prevention measures. There also exists an increased need to elucidate the trajectory of disease progression, from bacteremia to sepsis and ultimately, septic shock or complications such as meningitis or encephalitis. Given the systemic nature of sepsis and its widespread physiological impact, molecular approaches to biomarker discovery hold considerable promise. Multilayered molecular analyses encompassing the microbiome, metabolome, transcriptome, and proteome offer a systems level perspective revealing complex biological interdependence. Although numerous systematic reviews have examined the individual contributions of omics technologies towards the enhancement of sepsis diagnostics and a broader understanding of pathophysiology, integrative multi-omics investigations remain limited (20–22). Two reviews published as recently as 2018 have addressed multi-omic approaches; however, they do not account for the most recent advancements in molecular diagnostic techniques (22, 23). We conducted a scoping review of molecular studies published over the past five years with the aim of updating and refining our understanding of the diagnostic and staging landscape of neonatal sepsis. This review seeks to address existing knowledge gaps by synthesizing recent findings related to the application of molecular methodologies in the diagnosis, classification and management of neonatal sepsis. Specifically, we sought to address the following research question: How can insights derived from recent non-animal, original research molecular studies be synthesized or compared against each other to inform the identification of biomarkers that enhance the diagnosis, classification, and therapeutic management of neonatal sepsis?

### 1.1 Background knowledge of molecular studies in neonatal sepsis

#### 1.1.1 Microbiome

The neonatal microbiome has emerged as a critical area of research in early-life health and disease, with most studies to date focusing predominantly on the gastrointestinal tract, and to a lesser extent, the nasal microbiome. Understanding microbial colonization patterns and community dynamics in neonates is essential, given their potential influence on immune development, disease susceptibility, and clinical outcomes such as necrotizing enterocolitis (NEC) and sepsis. In a 2015 review, Gritz et al. (24) explored the association between neonatal gut dysbiosis and the development of NEC, with additional focus on the therapeutic potential of probiotics. While the review highlighted the significance of early microbial colonization patterns, it found no conclusive evidence supporting the efficacy of probiotics in reducing the incidence of either neonatal EOS or LOS (24).

Microbiome characterization in neonates typically relies on sequencing techniques. Two commonly employed approaches include 16S ribosomal RNA (rRNA) gene sequencing and shotgun metagenomic sequencing. In 16S rRNA sequencing, hypervariable regions of the bacterial 16S rRNA gene are selectively amplified from extracted DNA, passed through bioinformatics processing, and matched against curated microbial genome databases for taxonomic assignment. Shotgun metagenomic sequencing provides a more comprehensive approach by analyzing the entire genomic content of a sample, including bacterial, viral, fungal, and protozoan DNA. In this method, all extracted DNA is processed and subsequently annotated against genomic databases, enabling both taxonomic identification and functional profiling of microbial communities.

To quantify and compare microbial communities, several indices are available, each emphasizing different aspects of diversity. Alpha diversity refers to the richness and evenness of microbial species within a single sample. Indices measuring alpha diversity include the Shannon index, which measures both species richness and abundance, and the Inverse Simpson index, which places greater weight on the dominant species present. Beta diversity, in contrast, assesses compositional differences between microbial communities across distinct samples or groups. Key beta diversity metrics include the Bray-Curtis Dissimilarity, which reflects differences in species abundance, the Jaccard Index, which only considers the presence or absence of species, and the UniFrac, which incorporates phylogenetic relationships among taxa.

#### 1.1.2 Genomics

Genomic analysis involves comprehensive profiling of the entire genome to identify genetic variants associated with disease susceptibility. This goal is commonly achieved through whole genome sequencing (WGS), which interrogates the complete DNA sequence, or whole exome sequencing (WES), which targets only the protein-coding regions of the genome. In the context of neonatal sepsis, genomic studies conducted prior to 2022 (25) predominantly aimed to identify genetic markers associated with an increased susceptibility to infection. These markers form the basis for polygenic risk scores (PRS), which may enable early identification of neonates at increased risk of sepsis and related complications.

The insights gained from genomic profiling hold promise for the future of personalized medicine, including potential applications in gene therapy and genome editing technologies. The emergence of personalized medicine may also offer a targeted alternative therapy to conventional antibiotic treatment. However, several limitations currently constrain the broader implementation of genomic analysis in neonatal care. These limitations include the high financial cost of sequencing technologies, the complexity and volume of genomic data, and significant ethical concerns related to data ownership, consent, and long-term privacy of genetic information.

#### 1.1.3 Transcriptomics

Transcriptomics refers to the comprehensive analysis of the transcriptome—the complete set of RNA transcripts produced by the genome under specific physiological or pathological conditions. By profiling gene expression patterns, transcriptomic analysis provides insight into cellular function, disease states, and host responses to infection. Several methodologies are employed in transcriptome profiling, including microarray analysis, which quantifies predetermined messenger RNA (mRNA) sequences using complementary nucleotide probes. Other methodologies include bulk RNA sequencing, which measures average gene expression across a mixed population of cells, and single-cell RNA sequencing, which enables detection of gene expression at the level of individual cells and reveals heterogeneity within complex tissues (26).

Prior to 2018, transcriptomic applications in neonatal sepsis research focused on distinguishing neonates with sepsis from healthy controls to improve diagnostic precision (23). These studies aimed to identify host-derived transcriptional biomarkers that could differentiate between infectious and non-infectious inflammatory responses in early infancy. Unfortunateuly, widespread use of transcriptomic tools is limited by methodological and technical constraints. Microarrays are restricted to known gene sequences and are dependent on the availability of specific probes, necessitating prior knowledge of the organism's genome (23). RNA sequencing technologies, while more comprehensive, are hindered by the inherent fragility of RNA molecules, which require rapid sample processing and preservation (23). Furthermore, transcriptomic analyses generate large-scale datasets that demand robust bioinformatic infrastructure and computational resources for processing, storage, and interpretation (23).

#### 1.1.4 Mirnomics

MicroRNAs (miRNA) are small, non-coding RNA molecules typically 18–25 nucleotides in length that bind to complementary sequences on mRNA transcripts, thereby inhibiting translation or promoting mRNA degradation. These regulatory molecules are found in both the cytoplasm and circulating blood, and are key modulators of immune signaling pathways and cellular stress responses.

MiRNA profiling is often considered a specialized subset of transcriptomics, with both fields employing similar analytical platforms, but can also be studied independently as miRNomics. Technologies commonly used include microarrays, quantitative reverse transcription PCR, and small RNA sequencing, which allows for comprehensive detection of miRNA without prior sequence knowledge. miRNAs demonstrate increased molecular stability relative to mRNAs, making them attractive biomarker candidates in conditions such as neonatal sepsis. However, challenges remain particularly with microarray-based detection, as it requires predefined sequence probes.

MiRNA-focused studies conducted prior to 2018 have identified multiple differentially expressed miRNAs during episodes of neonatal sepsis. Many of these miRNAs have been implicated in innate immune system modulation, suggesting their potential utility in elucidating the molecular mechanisms underlying neonatal immune responses, and in contributing to the development of sepsis-specific biomarkers (23).

#### 1.1.5 Epigenomics

Epigenetics refers to the study of heritable changes in gene expression that occur without alterations to the underlying DNA sequence. These modifications regulate genomic activity and are influenced by a variety of endogenous and exogenous factors, including age, diet, pharmacologic agents, and environmental exposures. Epigenetic mechanisms include DNA methylation, histone modification, and regulation by non-coding RNAs, all of which contribute to chromatin remodeling and transcriptional control. Among these mechanisms, DNA methylation is the most extensively studied in the context of neonatal disease, as the process plays a key role in cellular differentiation, immune regulation, and disease susceptibility. The gold standard technique for methylation analysis involves bisulfite treatment, followed by DNA amplification and microarray hybridization to quantify levels of methylation with fluorescent signal intensity (27).

Epigenome-wide association studies have been conducted to investigate differential methylation patterns in neonates with and without sepsis (25). These studies have identified distinct epigenetic signatures associated with neonatal sepsis, suggesting aberrant DNA methylation in specific genes may serve as potential biomarkers for early detection or risk stratification in this vulnerable population.

#### 1.1.6 Proteomics

Proteomics, the large-scale study of the complete set of proteins expressed by an organism, provides crucial insight into cellular function and disease processes. Methods include targeted assays, such as enzyme-linked immunosorbent assays (ELISA), to measure individual proteins or small panels with a high specificity. In contrast, comprehensive or untargeted proteomic analyses are typically conducted using mass spectrometry (MS), which allows for a broad assessment of protein abundance across the proteome.

Prior to 2018, proteomic studies in neonatal sepsis primarily aimed to construct biomarker panels to diagnose sepsis and distinguish it from related conditions, such as necrotizing enterocolitis. Research from this time showed untargeted MS-based approaches to identify proteins significantly increased the likelihood of discovering novel biomarkers. Despite this potential, incorporation of proteomic analysis into point-of-care testing remains challenging. Key limitations include the high cost of instrumentation and the labor-intensive nature of sample processing and data analysis. These factors currently hinder the routine clinical application of proteomic methods for rapid bedside diagnostics (23).

#### 1.1.7 Metabolomics

Metabolomics is the comprehensive study of small-molecule metabolites, such as amino acids, glucose, lipids, and fatty acids, that reflect the dynamic physiological state of an organism. In the context of neonatal sepsis, metabolomics aims to characterize the host's metabolic response to infection and potential interactions with the infectious agent. Metabolomic analyses are typically conducted using nuclear magnetic resonance (NMR) spectroscopy or MS, often in combination with liquid chromatography (LC) or gas chromatography (GC). These technologies allow for the detection and quantification of a wide range of metabolites in biological fluids, such as serum and urine.

Studies conducted prior to 2018 demonstrated significant differences in metabolic profiles between neonates with and without sepsis (23). Specifically, increased concentrations of glucose and lactate were observed in patients with sepsis, alongside altered regulation of key metabolic pathways. Several metabolites identified in these studies have shown promise as novel biomarkers for the early detection or stratification of neonatal sepsis. However, their translation into clinical diagnostics remains unrealized (23).

The primary limitations of metabolomic profiling are seen particularly when using MS-based approaches and include the high cost of instrumentation, technical complexity, and time-intensive nature of analysis (23). These barriers currently preclude widespread implementation in routine neonatal care, although ongoing technological advancements may improve accessibility and feasibility in the future.

## 2 Methods

**Figure 1:**
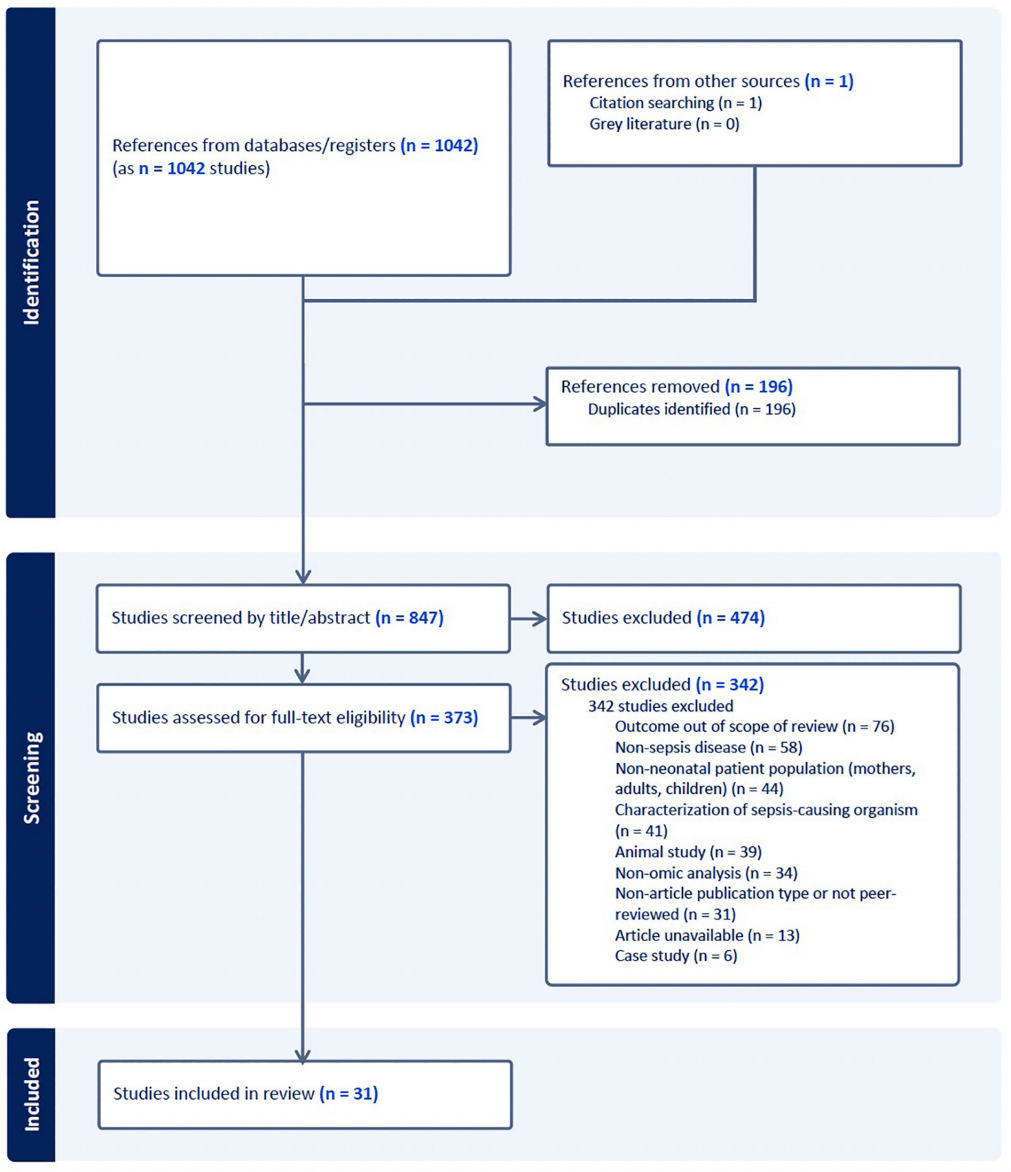
Summary of article screening process.

A systematic search of PubMed, EMBASE, and SCOPUS was conducted for articles published between January 2018 and June 2024 using the MeSH terms “neonatal sepsis”, “newborn”, “genomic*”, “proteomic*”, “transcriptomic*”, “microbiome”, “metabolomic*”, and “epigenomic*”. Equivalent controlled vocabulary terms were used for EMBASE and SCOPUS. Studies were included if they involved neonates aged 0–90 days with clinical suspicion or diagnosis of EOS or LOS, were published in English, and employed at least one molecular analysis technique as a primary methodology. Exclusion criteria included animal studies, non-original research, and dissertations. A total of 847 unique articles were identified, of which 31 met inclusion criteria for final review (Figure 1).

## 3 Results

### 3.1 Microbiome

Eleven studies investigating the role of the microbiome in neonatal sepsis were identified, primarily targeting gut and nasal microbiota to explore the influence of microbial colonization patterns and antibiotic exposure on sepsis development (Table 1). All but one study collected microbiome samples both before and after sepsis onset, enabling analysis of dysbiosis preceding infection. The exception, conducted by Pammi et al. (28), analyzed microbial profiles on removed central line catheters and skin swabs from neonates with and without central line-associated bloodstream infections (CLABSI).

**Table 1:** Summary of microbiome study characteristics and findings.

Reference	Study population and case definitions	Type	Methodology	Sample source	Brief summary of findings
Chang, et al.	120 neonates born preterm with gestational age between 24 and 28 weeks	Prospective cohort study	16s rRNA sequencing	Fecal samples	Early administration of probiotics may reduce the risk of LOS, potentially due to the increased abundance of Lactobacillus in the guts of infants who received supplementation with probiotics.
Graspeutner, et al.	71 infants born preterm with LOS and 164 neonates born preterm without sepsis between gestational age 23 and 33 weeks LOS defined as the first episode of clinical or culture-proven sepsis occurring after the first 72 h of life.	Prospective case-control study	16s rRNA sequencing	Fecal samples	Samples with LOS feature increased Bacilli species, less exclusively anaerobic species, less phylogenetic diversity. These characteristics may put neonates with LOS at greater risk for bacterial translocation due to increased gut permeability from increased metabolites that are fermentation products.
Lee, et al.	10 infants born preterm with very low birth weight (VLBW) and sepsis, 22 infants born preterm with VLBW without sepsis, 10 infants born at term without sepsis. Sepsis defined as presence of an invasive bacterial infection.	Prospective case-control study	16s rRNA sequencing	Fecal samples	Infants born preterm with WLBW and sepsis had lower microbial diversity in the gut at birth compared to infants born preterm without sepsis and infants born at term. PCR with strain-specific primers suggests translocation of bacteria from the gut to the bloodstream in certain cases. Prolonged antibiotic exposure significantly reduced beneficial Bifidobacterium and Lactobacillus in the gut.
Liu, et al.	24 infants born preterm, 4 of which developed necrotizing entercolitis and 3 of which developed LOS over the duration of the study. LOS defined as: – Positive hemoculture or other suspicious loci of infection after 72 h of life, – Clinical sepsis diagnosied by at least two neonatologists with response to advanced antibiotics	Prospective cohort study	16s rRNA sequencing	Fecal samples	Neonates with LOS were found to have greater abundance of Bacillus species, decreased diversity, increased deviation in phylogenetic similarity compared to healthy preterm control. All neonatal gut findings were present before onset of LOS.
Li, et al.	13 infants born to mothers positive for Group B Strep (GBS) and 73 infants born to mothers negative for GBS. GBS culture was conducted using vaginal swabs taken at 36 weeks gestational age for term labor or prior to delivery for premature labor.	Prospective case-control study	16s rRNA sequencing	Fecal samples	Infants with mothers positive for GBS have lower level of Lactobacillus species, which is a trend observed in neonates with EOS.
Mukopadhyay, et al.	18 infants at low risk for EOS (LRE) and 30 infants at non-low risk for EOS (non-LRE). LRE defined as delivery by cesarean section, absence of labor, and rupture of membrane at the time of delivery.	Prospective cohort study	WGS with shotgun metagenomic sequencing	Fecal samples	Infants with LRE show lower bacterial acquisition compared to infants with non-LRE in the first 3 days of life. A predominance of Staphylococcus epidermidis was observed in infants with LRE and Enterobacteriaceae sp. in infants with non-LRE between 4 and 7 days of life. 8 of the 9 cases of LOS detected over the duration of the study occurred in infants with non-LRE.
Pammi, et al.	15 neonates with central line-associated bloodstream infection (CLABSI) and 15 neonates without CLABSI. CLABSI was defined per the US Centers for Disease Control and Prevention criteria.	Prospective case-control study	16 rRNA sequencing	PICC catheter samples, skin swab samples, blood samples	The microbial DNA load was higher from catheter biofilms of neonates with CLABSI without differences in alpha diversity when compared to that of the neonates without CLABSI. Proteus and unclassified Staphylococcaceae were more abundant in infected catheter biofilms while Bradyrhizobium, Cloacibacterium, and Sphingomonas were more abundant in the uninfected catheters.
Schwartz, et al.	19 infants born preterm with bloodstream infection and 37 infants without bloodstream infection	Retrospective case-control study	WGS, shotgun metagenomic sequencing	Fecal samples	Significantly increased relative abundance of the causative species was noted in the microbiome in the 2 weeks preceding BSI for all cases vs. controls. The same BSI-causing species was found in stool before BSI onset in a subset of infants.
Wandro, et al.	32 infants with VLBW, 3 of which developed NEC, 8 developed LOS, and 21 remained healthy.	Retrospective cohort study	16s rRNA sequencing, GC-MS	Fecal samples	Bacterial abundance was decreased in neonates with LOS compared to controls. Bacterial communities were composed of mostly Enterobacteriaceae, Enterococcus, Staphylococcus, and Bacteroides organism. Increased fatty acids, lipid metabolism, and amino acids were positively correlated with the commonly abundant gut colonizers Enterobacteriaceae and Bacteroides and negatively correlated with the common low-abundance colonizers Staphylococcus and Enterococcus.
Zhao, et al.	43 neonates that acquiared S. aureus colonization and 82 neonates that did not acquire S. aureus colonization. Colonization was defined as growth on sheep blood agar and selective agar plates.	Case-control study	16s rRNA sequencing	Nasal swab samples	Lower biodiversity, uneven distribution of microorganisms, and S. aureus domination may increase risk of infection in neonates and serve as a biomarker for predicting disease/bacteremia in neonates.
Zhou, et al.	98 pregnant mothers and neonates divided into four groups: full term without antibiotic exposure, full term with antibiotic exposure, preterm without antibiotic exposure, and preterm with antibiotic exposure.	Prospective cohort study	16 s rRNA sequencing	Maternal vaginal swabs and fetal meconium samples	High loads of Bifidobacterium and Staphylococcus spp. in the gut microbiota as well as individual-specific vaginal microbiota of the 4 neonates that developed EOS over the course of the study.

A common finding across multiple studies was a reduction in alpha diversity in neonates with sepsis (Table 1), although the specific bacteria noted to be present or absent varied by study. Lee et al. (29) reported significantly lower Shannon index values in infants born preterm with very low birth weight (VLBW) and sepsis compared to infants born preterm and term with VLBW but without sepsis from birth through 4–7 weeks of age. In contrast to infants born at term, whose microbiomes showed increased *Firmicutes* and *Actinobacteria*, healthy infants born preterm exhibited greater levels of *Proteobacteria* (29) (Table 2). Liu et al. (30) observed reduced alpha diversity during EOS or LOS, while Graspeuntner et al. (31) identified a predominance of Bacilli class species, including *S. epidermidis* and *S. haemolyticus*, in neonates with sepsis (Table 2). Similarly, Zhao et al. (32) found decreased diversity with a *Staphylococcus aureus* dominance in the nasal microbiome of infants with LOS in the week preceding bacteremia (Table 2). Wandro et al. (33) also reported a dominance of *Staphylococcus* in fecal samples from neonates who developed LOS (Table 2).

**Table 2:** Taxonomic classification of bacteria studied with relevant findings. Blank cells indicate lower levels of classification not studied by the authors.

Phylum	Class	Genus	Species	Gram staining	Oxygen dependence	Result	Article(s)
Proteobacteria				Gram positive	Facultative anaerobic	Increased in dysbiosis of neonates born preterm	Lee, et al.
	Gammaproteobacteria	*Enterobacter*	*E. hormaechei*	Gram negative	Facultative anaerobic	Increased in microbiome during LOS	Graspeuntner, et al.
						Bloodstream infection more likely to occur with previous exposure to antibiotics	Schwartz, et al.
						Increased abundance during LOS	Wandro, et al.
						Increased in infants at non-low risk for EOS compared to infants at low risk	Mukopadhyay
		*Klebsiella*	*K. oxytoca*	Gram negative	Facultative anaerobic	Increased in microbiome during LOS	Graspeuntner, et al.
		*Proteus*		Gram negative	Aerobic	Increased abundance in neonates with central line-associated bloodstream infection	Pammi, et al.
		*Veillonella*		Gram negative	Anaerobic	Increased in microbiome during LOS	Graspeuntner, et al.
		*Yersiniaceae*		Gram negative	Facultative anaerobic	Slightly decreased with probiotics	Chang, et al.
		*Bacterioides*		Gram negative	Anaerobic	Increased abundance with LOS	Wandro, et al.
Actinobacteria	Actinobacteria	*Bifidobacteria*		Gram positive	Anaerobic	Increased in microbiome during LOS	Graspeuntner, et al.
						Increased with probiotics, but not significantly	Chang, et al.
						Increased in microbiome during EOS	Zhou, et al.
						Decreased with use of antibiotics	Lee, et al.
Firmicutes				Gram positive	Facultative anaerobic	Decreased in dysbiosis of neonates born preterm	Lee, et al.
	Bacillus	*Enterococci* (genus)		Gram negative	Facultative anaerobic	Dominant in microbiome before onset of LOS, decreased during LOS	Graspeuntner, et al.
		*Lactobacillus*		Gram positive	Facultative anaerobic	Significantly increased with probiotics	Chang, et al.
						Decreased with antibiotic exposure	Lee, et al.
						Decreased in infants with mothers positive for GBS	Li, et al.
		*Staphylococcus*	*S. epidermidis*	Gram positive	Aerobic or facultative anaerobic	Increased abundance during LOS	Graspeuntner, et al.
						Increased in infants at non-low risk for EOS compared to infants at low risk	Mukopadhyay
			*S. haemolyticus*			Increased abundance during LOS	Graspeuntner, et al.
						Slightly decreased with probiotics	Chang, et al.
						Increased in microbiome during EOS	Zhou, et al.
						Increased abundance with LOS	Wandro, et al.
						Increased abundance in neonates with central line-associated bloodstream infection	Pammi, et al.
		*Bacillales*		Gram positive	Aerobic or facultative anaerobic	Increased abundance with LOS	Graspeuntner, et al.
		*Bacillus*		Gram positive	Aerobic or facultative anaerobic	Increased in LOS	Liu, et al.
		*Streptococci*		Gram positive	Facultative and obligate anaerobic	Dominant in microbiome before onset of LOS, decreased during LOS	Graspeuntner, et al.

Several studies assessed the impact of antibiotics and probiotics on the neonatal gut microbiome (Table 1). Chang et al. (34) found increased *Lactobacillus* and *Bifidobacterium* in infants exposed to probiotics, though only *Lactobacillus* remained significant after adjusting for gestational age. Conversely, use of antibiotics resulted in reductions of the two genera in neonates with WLBW and EOS or LOS, as reported by Lee et al. (29). In a similar vein, Zhou et al. (35) report mothers who received perinatal antibiotics gave birth to neonates with reduced *Lactobacillus* in their meconium samples. Li et al. (36) also noted lower *Lactobacillus* abundance in neonates born to Group B Streptococcus (GBS)-positive mothers. In regards to levels of *Bifidobacterium,* Wandro et al. (33) observed an absence in fecal samples of neonates born preterm with LOS, likely due to uniform antibiotic exposure.

Other findings on the impact of antibiotics include those reported by Mukhopadhyay et al. (37), namely a predominance of *Enterobacteriaceae* in fecal samples from infants with VLBW exposed to antibiotics during the first week of life, though the relationship with subsequent bacteremia was not specified. In contrast, Schwartz et al. (38) found that neonates with bloodstream infections caused by *Enterobacteriaceae* were more likely to have received antibiotics within 10 days of infection onset. Mukhopadhyay et al. (37) also noted an increasing trend in *Enterobacteriaceae* dominance over time, regardless of antibiotic exposure. However, no consistent shifts in relative abundance of the causative pathogens were observed in cases with LOS before or after antibiotic treatment, and infants generally remained clinically stable following discontinuation of antibiotics (37).

### 3.2 Genomics

Three genomic studies published between 2018 and 2024 were included in the final review, each applying whole genome sequencing technologies with distinct objectives (Table 3). Two studies, conducted by Lipworth et al. (39) and Sethi et al. (40), focused on genomic characterization of bacterial isolates responsible for neonatal sepsis, while Ciesielski et al. (41) applied host genome sequencing to identify genetic variants associated with LOS in neonates.

**Table 3:** Summary of genomic study characteristics and findings.

Reference	Study population and case definitions	Type	Methodology	Sample source	Brief summary of findings
Ciesielski, et al.	233 neonates with LOS and 288 neonates without LOS LOS defined as positive blood culture or abnormal clinical and laboratory findings.	Case-control genome-wide association study	Illumina MEGA Consortium V2 Beadchip SNP analysis, pathway analysis	Blood samples	There appears to be a connection between LOS and IL10 and two uncharacterized genes on chromosomes 16 and 6, and genes involved in NOTCH signaling may be involved in distinguishing sepsis by sex of patient.
Lipworth, et al.	327 isolates of gram-negative blood stream infection in individuals under 18 years of age, 124 of which were found in neonates	Retrospective cohort study	Illumina HiSeq 2,500/3,000/4,000/MiSeq instruments	Blood samples	O-antigen targeted vaccines show potential to reduce indicence of E. coli bloodstream infections in neonates
Sethi, et al.	1,274 staphylococci isolates from neonates admitted to two NICUs over a 10-week period	Cohort study	Illumina Tagment DNA Enzyme and Buffer	Ear, nose, groin, rectum swabs from one NICU. Axilla, groin, rectum, throat swabs from the second NICU. One blood culture and two wound swabs from the second NICU.	No significant phylogenetic links were found between antibiotic resistance in staphylococci associated with chlorhexidine gluconate usage.

In the study by Ciesielski et al. (41), single nucleotide polymorphism (SNP) analysis was used to explore host genetic susceptibility to LOS. Distinct autosomal and X-linked SNPs were identified in male and female neonates with no overlapping variants between sexes (41). Several of the SNPs associated with LOS were mapped to genes involved in NOTCH signaling, a pathway implicated in T-cell regulation and apoptosis. Furthermore, five SNPs associated with IL-10 and one with TNF-α were identified (41).

Lipworth et al. (39) conducted whole genome sequencing of *Escherichia coli* isolates from neonatal bloodstream infections. A notable finding was that a significant proportion of the isolates possessed O-antigen serotypes covered by the ExPEC-4V vaccine, currently under phase II clinical evaluation in adults (39).

Sethi et al. (40) investigated antiseptic susceptibility in neonatal sepsis isolates across hospitals in the UK and Germany. Using genomic sequencing, the study found higher minimum inhibitory concentrations (MICs) for chlorhexidine in UK isolates, where the antiseptic is more widely used, compared to German isolates (40). However, genomic analysis did not identify any significant associations between chlorhexidine or octenidine susceptibility and known resistance markers, including *qac* genes or NorA/NorB efflux pump genotypes (40).

During the broader screening process, an additional 55 studies were identified that utilized genomic sequencing for epidemiological investigation of NICU outbreaks. These studies primarily focused on identifying transmission patterns and outbreak sources, and while highly valuable for infection control, they were excluded from the final review due to limited applicability to biomarker discovery. A summary of these studies is provided in Table 4 for reference.

**Table 4:** Summary of characteristics of epidemiologic studies employing genomic analysis.

Author	Microorganism	Location	Setting	Purpose of omic analysis used
Afeke, et al.	Staphylococcus epidermidis	Ghana	NICU	Sequence the strains
Ali, et al.	Streptococcus agalactiae	Ethiopia	2 rural hospitals, 1 urban hospital	Investigate serotype distribution, clonal relationships, lineage distributions, virulence factor determinants, and antimicrobial susceptibility patterns
Baier-Grabner, et al.	Yersiniabactin-producing Klebsiella aerogenes	Austria	NICU	Identify allelic relatedness and pathogenicity of isolates
Bar-Meir, et al.	Bacillus cereus	Israel	tertiary NICU	Identify relatedness of clones isolated from samples
Bojang, et al.	Staphylococcus aureus	Gambia	Labour ward	Characterization of clone inheritance
Brinkac, et al.	Klebsiella quasipneumoniae	Nigeria	NICU	Trace origin of plasmid conferring resistance and identify phylogenetic relatedness of isolates
Cho, et al.	methicillin-susceptible Staphylococcus aureus	USA	Level 4 NICU	Clonal diversity of isolates and phylogenetic relatedness of isolates
Collins, et al.	Group B strep	UK and Ireland	NICU	Identify determine a single-nucleotide polymorphism (SNP) difference threshold of clustered cases
Cornick, et al.	Klebsiella pneumoniae	Malawi	NICU	Identify strain characteristics, resistance genes, and causative clone
Delettre, et al.	Group B strep	France	NICU	Identify similarities among isolates
Dos Santos, et al.	Carbapenem-producing Enterobacteriaceae	Gabon	Neonatology and maternal wards	gGin insights into the genetic diversity, virulome, resistome, and plasmid content of clinical and environmental CPE isolates
Ferry, et al.	Enterobacter cloacae	France	NICU	Identify phylogenetic relatedness of strains
Frenk, et al.	Extended-Spectrum Beta-Lactamase-Producing Klebsiella Pneumoniae	Israel	3 NICUs	Identify if outbreaks were results of plasmid dissemination between clones
Furfaro, et al.	Streptococcus agalactiae	Western Australia	NICU and Obstetrics unit	Characterize the strain local to Western Australia
Girlich, et al.	E cloacae	France	NICU	Decipher the transmission routes
Gramatniece, et al.	Acinetobacter baumannii	Latvia	NICU	Compare drug-resistant strains with strains identified in outbreak
Hallback, et al.	OXA-48-producing Enterobacteriaceae	Sweden	NICU	Strain typing and plasmid tracing using whole-genome sequencing for the analysis of the transmission of bacterial strains and *in vivo* transfer of plasmids
Haydecke, et al.	Escherichia coli	Sweden	NICU	Analyzed for virulence factors
Hernandez-Alonso, et al.	Enterobacter	France	NICU	Characterize Enterobacter strain gene content and to provide a comprehensive understanding of the epidemiological dynamics of the outbreak, identify phylogenetic relatedness of isolates
Hume, et al.	Staphylococcus capitis	Australia	2 NICUs	Genomic assessment of resistance genes and virulence factors and phylogenetic analysis to assess for presence of nosocomial transmission and to assess if related to internationally distributed multi-resistant NRCS-A clone
Jauneikaite, et al.	Group B strep	UK	Tertiary NICU	Determine phylogenetic relatedness of isolates, attempt to determine source of clusters
Kotsanas, et al.	Enterococcus faecalis	Melbourne	Tertiary care NICU	Identify and type isolates
Labi, et al.	Carbapenemase-Producing Klebsiella pneumoniae	Ghana	NICU	Identify phylogenetic relatedness, shared resistance genes, shared plasmids
Labi, et al.	carbapenemase-producing Klebsiella pneumoniae	Ghana	2 tertiary NICUs	Strain characterization
Mabena, et al.	MDR Enterobacter spp., Staphylococcus aureus, Klebsiella pneumoniae, Acinetobacter baumannii, Pseudomonas aeruginosa and Enterococcus faecium	South Africa	NICU	Identify similarities between colonizing and bloodstream isolates of bacteria in same child
Magnan, et al.	Staphylococcus haemolyticus	France	NICU	Sequence typing, phylogenetic relatedness
Magobo, et al.	Carbapenem-resistant Klebsiella pneumoniae	South Africa	NICU	Track transmission of clones
Mahony, et al.	Vancomycin-resistant Enterococcus faecium	Australia, New Zealand	NICUS and more	Identify route of transmission for multi-center outbreak, phylogenetic relatedness, resistance genes
Manandhar, et al.	mcr-10-carrying Enterobacter kobei	Nepal	tertiary NICU	Identify phylogenetic relatedness of isolates
Marando, et al.	Klebsiella pneumoniae	Tanzania	NICU	Sequence typing to identify clonal origins of isolates
McGee, et al.	Group B strep	USA	Isolated submitted to CDC	Characterize the isolated
Moore, et al.	Staphylococcus capitis NRCS-A	UK	4 NICUs	Confirm identity of isolates as clone NRCS-A
Morhart, et al.	Enterobacter Hormaechei	Germany	level 3 NICU	Identification of the phylogenetic relationship and potential antimicrobial resistance genes, idenfy the source ECC that started the outbreak
Mukherjee, et al.	Klebsiella pneumoniae	India	Tertiary NICU	Phylogenetic relatedness, virulence factors, characterization
Muldermans, et al.	Serratia marcescens	Belgium	NICU	Identify relatedness of clones isolated from samples
Nieto-Rosado, et al.	Various gram negative bacteria	Bangladesh, Ethiopia, India, Nigeria, Pakistan, Rwanda, South Africa	NICU amongst other wards	Determine relatedness of species to those causing neonatal sepsis, identify transmission routes
Nievas, et al.	Elizabethkingia anophelis	Argentina	NICU	Confirm presence of outbreak
Nordberg, et al.	Gram Neg bacilli	Sweden	4 NICUs and 6 delivery units	Identify indicence, rates of AMR development
Nurjadi, et al.	Cephalosporin-resistant Enterobacterales	Germany	tertiary NICU	Molecular characterization and transmission analysis
Okomo, et al.	B cepacia and extended spectrum *β*-lactamase (ESBL)-producing Klebsiella pneumoniae	Gambia	NICU	Identify relatedness of clones isolated from samples and identify the timeline of outbreaks
Otto, et al.	Human Adenovirus Type 56	USA	3 NICUs	Genetic characterization of three new strains
Perez-Palacios, et al.	Enterobacterales	Morocco	NICU	Characterize the resistome; multi-locus sequence typing was used to investigate phylogeny
Perlaza-Jimenez, et al.	Klebsiella quasipneumoniae	China	NICU	Identify isolate diversity, resistance genes, virulence factor genes
Portal, et al.	Various gram negative bacteria	Nigeria	NICU and maternal wards	Characterization of mcr gene
Sands, et al.	Various bacteria	Bangladesh, Ethiopia, India, Nigeria, Pakistan, Rwanda, South Africa	NICU amongst other wards	Characterization of isolates, intra and interspecies diversity, antibacterial resistance genes
Sbaa, et al.	Group B strep	France	NICUs and more	Identify any differences between straings causing recurrent neonatal infections and nonrecurrent infections
Shi, et al.	Extended-spectrum β-lactamase-producing Klebsiella pneumoniae	China	NICU	Determine phylogenetic relatedness of isolates, identify transmission routes
Stenmark, et al.	Staphylococcus capitis	Sweden	University hospital (neonates)	Determine cluster relatedness of isolates
Strysko, et al.	Carbapenem-resistant Acinetobacter baumannii	Botswana	NICU and ICU	Identify relatedness of clones isolated from samples and identify the route of transmission
Sullivan, et al.	Methicillin-Resistant Staphylococcus aureus	US	NICU	Spatiotemporal characterizatin of outbreak progression
Tack, et al.	Salmonella Typhi	DR Congo	NICUs amd more	Identify antibiotic resistance genes and susceptibility genes
Toleman, et al.	methicillin-resistant Staphylococcus aureus	England	NICU	Genomic surveillance
van Kassel, et al.	Group B strep	Netherlands	NICUs and more	Serotype identification
Westberg, et al.	Staphylococcus haemolyticus	Sweden	NICU	Identify relatedness of clones isolated from samples
Wisgrill, et al.	Yersiniabactin-producing Klebsiella pneumoniae	Austria	NICU	Confirm presence of outbreak

### 3.3 Transcriptomics

Six studies published between 2018 and 2024 utilized transcriptomic technologies as the primary method for investigating neonatal sepsis and were included in this review (Table 5). The studies demonstrated considerable heterogeneity in focus and design, including comparisons of neonates with EOS vs. LOS vs. neonates without sepsis (42), gram-positive vs. gram-negative infections (23), and varying clinical outcomes such as bacteremia vs. septic shock (43). No two studies directly evaluated the same neonatal subgroups, limiting direct comparisons across investigations.

**Table 5:** Summary of transcriptomic study characteristics and findings.

Reference	Study population and case definitions	Type	Methodology	Sample source	Brief summary of findings
Bai et al. (44)	159 infants, with EOS (*n* = 60) and non-infected (*n* = 99) EOS defined as: – Significant clinical symptoms of sepsis (respiratory distress, anemia, fever, decreased absolute neutrophil count, or increased C-reactive protein) – Postive blood culture	Retrospective case-control study	mRNA microarray analysis, qRT-PCR	Peripheral blood	Identified 4 genes to be used in a diagnostic model for EOS: CST7, CD3G, CD247, and ANKRD22.
Cernada et al. (45)	55 infants born preterm with WLBW and Gram-positive EOS or LOS (*n* = 17), Gram-negative EOS or LOS (*n* = 8), and non-infected (*n* = 30) Sepsis defined as: – At least 3 of the following clinical signs: temperature stability, respiratory symptoms, cardiovascular symptoms, tachycardia, bradycardia, neurological symptoms, gastrointestinal symptoms. – Two positive blood cultures for coagulase negative sepsis	Prospective, observational, double-cohort study	RNA microarray analysis with over 750,000 probes, RT-PCR	Peripheral blood	Identified 8 genes differentially overexpressed in Gram-positive sepsis compared to Gram-negative sepsis: CD37, CSK, MAN2B2, MGAT1, MOB3A, MYO9B, SH2D3C, and TEP1. Identified most significantly overexpressed pathways in Gram-positive sepsis as relating to metabolism and immune response that regulate anti- and pro-inflammatory responses.
Huang et al. (43)	335 infants less than 6 months in age, with bacterial sepsis (*n* = 151), bacterial sepsis with septic shock response (*n* = 30), and non-infected (*n* = 154) Sepsis definitions varied between each original study used in the secondary analysis, with consensus of a positive blood culture	Secondary data analysis	Normalization of datasets	Peripheral blood	Identified differentially expressed genes that depict disease progerssion from bacterial sepsis to a septic shock response. Suppressed lymphocyte activity, induction hemostatic processes, and heightened innate immunity were all implicated in the transcriptomic profile of septic shock compared to bacterial sepsis and non-infected cases.
Ng et al. (78)	18 infants born very preterm with confirmed LOS (*n* = 5), possible LOS (*n* = 4), or no LOS (*n* = 9) LOS defined as: – Positive blood culture – Elevated C-reactive protein levels	Retrospective case-control study	PolyA-enrichement of RNA	Peripheral blood	Identified transcriptional alterations that distinguish confirmed LOS cases from non-infected cases as mainly involving pathways relatuing to TLR signaling, pro- and anti-inflammatory cytokine signaling, immune regulation, and altered cholesterol metabolism. Unable to distinguish infants with possible LOS from infants with no LOS or confirmed LOS.
Qi et al. (62)	GSE54514 Dataset: 53 blood samples, with sepsis survivors (*n* = 26), nonsurvivors (*n* = 9), and healthy non-infected controls (*n* = 18) GSE3140 Dataset: 12 samples, with healthy adult volunteers (*n* = 6), and healthy, full-term infants (*n* = 6)	Secondary data analysis	mRNA expression sequencing, differential expression gene analysis	Cord and peripheral blood	Identified upregulation of IFN-1 signaling in nonsurvivors of neonatal sepsis. Identified upregulated pathways in nonsurvivors of neonatal sepsis as including “type I interferon signaling pathway”, “cellular response to type I interferon”, “response to type I interferon”, “negative regulation of viral genome replication” and “regulation of viral genome replication”.
Sweeney et al. (79)	213 infants compiled from 3 original studies, with sepsis (*n* = 103) and non-infected (*n* = 110) Sepsis defined by the original studies	Secondary data analysis	Microarray normalization of datasets	Peripheral blood	Identified that the Sepsis MetaScore, which constructs a gene-expression-based signature, outperformed standard laboratory measurements alone for neonatal sepsis

While the heterogeneity in patient populations and research aims limited the identification of consistently reported individual genes, *CD3G* emerged as a noteworthy exception (Table 6). This gene, which plays a key role in T-cell receptor signaling, was reported as downregulated in both Bai et al. (44) and Huang et al. (43). Bai et al. (44) highlighted *CD3G* as one of four differentially expressed genes distinguishing neonates with EOS from neonates without sepsis, while analysis by Huang et al. (43) revealed *CD3G* as a key gene differentiating neonates with septic shock from those with bacteremia.

**Table 6:** Summary of genes of interest identified by transcriptomic analysis.

Gene function	Gene	Result	Article	Brief description of function/involvement in sepsis
Overall cell function and survival	MYO9B	Overexpressed in Gram-positive sepsis	Cernada, et al.	Promotes cell migration and differentiation
	BCL2	Down-regulated in LOS	Ng, et al.	Anti-apoptotic protein
	TEP1	Overexpressed in Gram-positive sepsis	Cernada, et al.	Promotes cell function
Kinase activity	MOBKL2A (MOB3A)	Overexpressed in Gram-positive sepsis	Cernada, et al.	No direct relationship found to inflammatory response, immune response, cell death, apoptosis, cell migration, cell differentiation, and metabolic response
	PI3KCA	Common marker hub gene of neonatal sepsis	Meng, et al.	Encodes p110 alpha (p110α) protein component of PI3K enzyme
Immune response (promoting)	MGAT1	Overexpressed in Gram-positive sepsis	Cernada, et al.	Trigger effective cell migration, inflammatory response, and immune response.
	CST7	Differentially expressed in EOS, upregulated	Bai, et al.	Encodes Cysteine F to regulate the cytotoxicity of natural killer (NK) cells within the tumor microenvironment
	CSK	Overexpressed in Gram-positive sepsis	Cernada, et al.	Promotes cell differentiation and migration in IR, levels can determine the strength of the IR. Overexpression may be anti-apoptotic.
	CD3G	Differentially expressed in EOS, downregulated	Bai, et al.	Typically upregulated in T cells, inversely correlated with sequential organ failure and mortality in sepsis
		Downregulated in bacteremic neonates compared to in septic shock or healthy neonates	Huang, et al.	
	CD37	Overexpressed in Gram-positive sepsis	Cernada, et al.	Leukocyte-specific protein of tetraspanin family, underexpression leads to poor IR but overexpressed in Gram pos sepsis. Overexpression may by anti-apoptotic.
	SH2D3C	Overexpressed in Gram-positive sepsis	Cernada, et al.	Regulator of lymphocyte adhesion in the integrin activation pathway.
	CXCL10	Upregulated in bacteremic infants whose transcriptomic profiles suggested a trend towards septic shock (upregulated when comparing septic shock to healthy)	Huang, et al.	Recruiting leukocytes, such as T cells, eosinophils, and monocytes, to sites of inflammation
	CKAP4	Upregulated in septic shock and downregulated in bacteremia	Huang, et al.	Regulation of cell adhesion sites and migration
	CD3E, ZAP70, LCK and CD5	Downregulated in bacteremia compared to septic shock		T-cell activation
	SERPINB10	Upregulated in septic shock infants and some bacteremic infants		Regulation of protease activities during hematopoiesis and apoptosis induced by TNF
	IL1R2	Upregulated in LOS	Ng, et al.	Immune inhibitory
	IL1RN	Upregulated in LOS		Immune inhibitory
	IL-7	Down-regulated in LOS		Promote growth and function of B-cells and T-cells
	IL-10	Upregulated in LOS		Attenuates immune response and promotes wound healing
	SOCS1	Upregulated in LOS		Suppressor of cytokine signaling
	SOCS3	Upregulated in LOS		Suppressor of cytokine signaling
	CD247	Downregulated in EOS	Bai, et al.	Involved in human and murine sepsis, involved in the occurrence and development of sepsis
	NFKBIA	Upregulated in LOS	Ng, et al.	Role in TLR signaling
	MYD88			
	CEBPB			
	STAT1			
	IRF7			
	IRAK2			
	IRAK4			
	TBK1			
Metabolism	LDHA	Upregulated in LOS	Ng, et al.	Encodes lactate dehydrogenase A, necessary for production of pyruvate
	SREBF2			Involved in cholesterol biosynthesis
	DHCR7			
	INSIG1			
	ERLIN2			
	SQLE			
	IDI1			
	LDLR			
	PPARG			Regulation of lipid and glucose metabolism
Mitochondrial function	ANKRD22	Upregulated in EOS	Bai, et al.	ANKRD22 is upregulated, encodes a specific mitochondrial protein, involved in progression of various cancers but connection to sepsis is unclear.
	CISD2	Distinguishes septic shock from bacteremia	Huang, et al.	Maintain mitochondrial function
Tissue repair	MM8	Highest in neonates with shock vs bacteremia	Huang, et al.	Directly correlated with mortality in sepsis

Although overlap in specific genes was rare, several studies identified genes involved in similar immune pathways**,** suggesting convergent biological processes (Table 6). Cernada et al. (45) reported upregulation of *CSK*, a gene implicated in immune cell differentiation and migration, in gram-positive compared to gram-negative sepsis. Huang et al. (43) identified *CXCL10*, a chemokine known for recruiting immune cells to infection sites, as significantly upregulated in septic shock vs. bacteremia.

A cross-study comparison of two studies revealed a potential shared inflammatory pathway as the subject of investigation (Table 6). Huang et al. (43) reported increased expression of *MM8*, a gene associated with severe inflammatory responses, in neonates with septic shock. Ng et al. (23), examining cases with LOS, found upregulation of NF-*κ*B1A, a downstream target activated by MM8. NF-*κ*B1A mediates production of key inflammatory cytokines such as IL-6 and type I interferons.

### 3.4 Mirnomics

Two studies employing miRNA sequencing were selected for review, both of which used a prospective case-control design (Table 7). Serna et al. (46) investigated infants with VLBW and LOS, while Janec et al. (47) focused on neonates born preterm with EOS. Both studies identified differentially expressed miRNAs in neonates with sepsis that are known regulators of innate immune responses. Janec et al. (47) reported increased expression of miR-142-5p, miR-223-3p, and miR-148b-3p in neonates with sepsis. These miRNAs are associated with suppression of Toll-like receptor 4 (TLR4) signaling, a key pathway in the detection of bacterial pathogens. Serna et al. (46) found under expression of several immune-regulatory miRNAs in neonates with sepsis. These included miR-23, which modulates innate immunity through regulation of metalloproteinase 10; miR-17, which is involved in broader immune responses including T-cell function; and miR-15a, which suppresses TLR4 and IRAK1 expression in response to lipopolysaccharide (LPS) exposure.

**Table 7:** Summary of miRNOmic study characteristics and findings.

Reference	Study population and case definitions	Type	Methodology	Sample source	Brief summary of findings
Serna, et al.	11 neonates born preterm with very low birth weight and gram-positive LOS and 16 healthy neonates without LOS Gram-positive sepsis defined as positive blood culture with gram-positive species	Prospective case-control study	Small non-coding RNA profiling	Blood samples	Cases of gram-positive LOS show under expression of miR-23, an important regulator of innate IR and under expression of miR-15a, which down-regulates expression level of TLR4 and IRAK1 induced by LPS.
Janec, et al.	8 neonates born preterm with EOS and 12 neonates without sepsis EOS defined by positive blood culture	Prospective case-control study	miRNA sequencing	Blood samples	Peripheral plasma of neonates with EOS shows upregulation in miRNA in inflammatory response.

### 3.5 Epigenomics

One study by Lorente-Pozo et al. (48) conducting epigenomic analysis was selected for review (Table 8). Neonates with sepsis show differential hypermethylation of the *CD3G* and *CD3D* gene promoters and hypomethylation of *IL10* gene promoter compared to controls without sepsis. Greater changes in methylation were found when comparing neonates with LOS to controls than comparing neonates with EOS to controls. When comparing methylation in neonates with LOS to neonates with EOS, 4 significantly hypomethylated gene promoters were noted- *DUSP22, PM20D1, MIR10A*, and *MIR886*. Pathway analysis revealed significant hypermethylation of regions involved in T-cell activation and T-cell differentiation, and hypomethylation of regions involved in neutrophil and mast cell activation when comparing neonates with sepsis to controls.

**Table 8:** Summary of miRNomic study characteristics and findings.

Reference	Study population and case definitions	Type	Methodology	Sample source	Brief summary of findings
Lorente-Pozo, et al.	23 neonates born preterm, 6 with confirmed EOS, 9 with confirmed LOS, 2 with confirmed EOS and LOS, and 6 neonates without sepsis. EOS defined as a positive peripheral smear culture, suggestive symptoms (temperature instability, cardiovascular symptoms, neurological symptoms, gastrointestinal symptoms or clinical suspicion), and/or increased plasma C-reactive protein or IL-6 levels, diagnosed within the first 72 h of life. LOS defined as a positive blood culture or suggestive clinical symptoms, diagnosed after the first 72 h of life.	Prospective cohort study	Genome-wide methylation profiling	Blood samples	Neonates with sepsis display significant hypermethylation of regions involved in T-cell activation and T-cell differentiation, and hypomethylation of regions involved in neutrophil and mast cell activation when compared to controls. When comparing EOS and LOS, neonates with LOS have hypomethylation of anti-apoptotic gene promoters

### 3.6 Proteomics

Two studies utilizing liquid chromatography–mass spectrometry (LC-MS) were selected for this review, each investigating different biological fluids to better understand protein expression in neonatal sepsis (Table 9). Chatziioannou et al. (49) conducted a prospective matched case-control study to evaluate the serum proteomic profile of neonates with LOS. Analysis of serum samples identified a panel of three proteins that showed potential as diagnostic biomarkers: apolipoprotein A-IV (APOA4), cholesteryl ester transfer protein (CETP), and C-reactive protein (CRP) (49). In contrast, Jiang et al. (50) explored cerebrospinal fluid (CSF) proteomic changes in a cohort of neonates with sepsis who also exhibited signs of neuroinflammation. Analysis of CSF samples revealed a consistent decrease in proteins essential for neuronal circuit formation and extracellular matrix (ECM) stability (50). Among these were matrix metalloproteinase-2 (MMP-2), an enzyme that degrades components of the ECM, and cystatin C, a protease inhibitor known to play a protective role in the central nervous system. Other proteins such as brevican, nidogen-1 and −2, and fibronectin, all important structural elements of the ECM, were also found to be reduced in neonates with neuroinflammatory responses (50).

**Table 9:** Summary of proteomic study characteristics and findings.

Reference	Study population and case definitions	Type	Methodology	Sample source	Brief summary of findings
Chatziioannou, et al.	25 neonates with necrotizing enterocolitis, 18 neonates with LOS, and 43 matched control neonates	Prospective case-control	LC-MS	Serum samples	Proteins CRP, CETP, and APOA4 is the optimal combination of proteins to use as biomarkers of LOS
Jiang, et al.	9 neonates with sepsis and neuroinflammation and 20 samples with sepsis without neuroinflammation Sepsis defined as two or more of a list of abnormal laboratory values. Neuroinflammation defined as at least one of the following: WBC count >20 × 106/L; glucose concentration <2.2 mmol/L; or protein concentration >1,700 mg/L	Prospective cohort	LC-MS	CSF samples	The proteins with differential abundance between the neuroinflammation and no neuroinflammation patients are involved in development and maturation of the nervous system as well as maintaining the extracellular matrix.

### 3.7 Metabolomics

Six studies were selected that employed mass spectrometry-based metabolomic profiling to investigate neonatal sepsis (Table 10). All but one (51) utilized LC-MS as the primary analytical method, with a single study relying on gas chromatography coupled with time-of-flight mass spectrometry (GC-TOF-MS). These studies highlighted distinct alterations in the metabolic landscape of neonates with sepsis, revealing potential biomarkers and underlying pathways involved in disease progression.

**Table 10:** Summary of metabolomic study characteristics and findings.

Reference	Study population and case definitions	Type	Methodology	Sample source	Brief summary of findings
Frerichs, et al.	121 neonates born preterm with LOS and 121 preterm neonates without LOS. LOS defined as satisfaction of three criteria: clinical signs of generalized infection, a positive blood culture at least 72 h after birth, and initiation of antibiotic treatment with the intention to treat for at least 5 consecutive days. Neonates must be diagnosed with LOS within the first 28 days of life.	Prospective matched case-control study	GC-MS, GC-TOF-MS	Fecal samples	Various hydrocarbons were found to be important for the discrimination of gram-negative LOS vs. matched controls. Heptanal was identified as the exclusive metabolite for the discrimination of Coagulase negative-Staphylococcus LOS. Observed differences were most evident in gram-negative LOS vs. controls three days to one day before onset.
Liu, et al.	18 neonates born preterm with LOS, 42 healthy neonates. LOS defined as: abnormal clinical manifestations at after the third day of life, including positive non-specific findings on blood examination, CSF changes suggestive of purulent meningitis, or positive blood culture	Prospective case-control study	LC-MS	Fecal samples	Changes in levels of N-Methyldopamine, cellulose, glycine, gamma-Glutamyltryptophan, N-Ribosylnicotinamide and 1alpha, 25-dihydroxycholecalciferol showed the greatest potential as non-invasive biomarkers for LOS.
Mardegan, et al.	15 neonates with EOS and 15 neonates without EOS EOS defined as the presence of clinical signs and laboratory findings obtained within 72 h of birth (leukopenia, leukocytosis, increased C-reactive protein, increased serum lactate)	Prospective matched case-control study	LC-MS	Urine samples	Infants with EOS show elevated level of tyrosine metabolism products and glutathione metabolism products. Pathways over-represented are related to amino acid metabolism.
Thomaidou, et al.	15 neonates with LOS, 17 neonates with NEC, and 16 control neonates without LOS or NEC. LOS defined using criteria defined in 2010 by an Expert Meeting of the European Medicines Agency on Neonatal and Pediatric Sepsis.	Prospective case-control study	LC-MS	Serum samples	LOS shows decreased phosphatidylcholines (PCs) and lysoPCs that are important for membrane structure, enzyme activation, and anti-inflammation. LysoPC (16:0/0:0) seems to be the most promising as a sepsis biomarker. Decreased PCs and lysoPCs also associated with increased mortality.
Wang, et al.	30 infants with signs of infection and confirmed sepsis and 30 neonates without infection Sepsis confirmed by tests including blood test and C-reactive protein.	Prospective matched case-control study	LC-MS	Serum samples	Metabolism of various lipids was upregulated in the sepsis group. Levels of sphingomyelin, prolylhydroxyproline, and phosphorylcholine were decreased in the sepsis group.
Zhang, et al.	42 neonates with sepsis and meningoencephalitis (MEN) and 42 neonates with sepsis without MEN Sepsis defined as positive blood culture, non-specific signs of infection, and detection of abnoramlities in blood inflammatory response markers. MEN defined as CSF culture positive for pathogens and abnormal neurological symptoms.	Retrospective cohort	LC-MS	CSF samples	Changes in the CSF and serum metabolomes of the group with meningoencephalitis were manifested mainly as changes in arginine metabolism, which were closely related to changes in creatinine metabolism, oxidative stress-related markers, and potentially harmful bile acid and aromatic compound metabolism.

Alterations in lipid metabolism emerged as a common finding (Table 11). Both Thomaidou et al. (52) and Wang et al. (53) reported disruptions in lipid-related pathways in neonates with LOS. Thomaidou et al. (52) found reduced levels of phosphatidylcholine (PC) and lysophosphatidylcholine (lysoPC), with one specific lysoPC species, identified as 16:0/0:0, standing out as a strong biomarker candidate. Importantly, decreased levels of these lipids were also associated with increased mortality (52). Wang et al. (53) expanded upon these findings, reporting decreased sphingomyelin, prolyl hydroxyproline, and phosphorylcholine in cases with LOS. Interestingly, they also observed elevated levels of cytidine 5′-diphosphocholine (CDP-CHO), a precursor in PC biosynthesis (53).

**Table 11:** Summary of metabolites of interest identified using metabolomic analysis.

Metabolite	Sample source	Result	Article
1alpha-25-dihydroxycholecalciferol	Stool	LOS sepsis < Control	Liu, et al.
14-carbon, 16-carbon, 18-carbon, 20-carbon saturated phosphatidylcholine (PC) or lysophosphatidylcholine (LysoPC)	Blood/serum	LOS sepsis < Control	Thomaidou, et al.
2,2,4,4-tetramethylpentane	Stool	Gram neg LOS > control	Frerichs, et al.
5-HIAA	Blood/serum	EOS sepsis > Control	Mardegan, et al.
ADMA	Blood/serum	EOS sepsis > Control	Mardegan, et al.
alanine	Blood/serum	EOS sepsis > Control	Mardegan, et al.
aminoadipic acid	Blood/serum	EOS sepsis > Control	Mardegan, et al.
arginine	CSF	Upregulated in sepsis with meningoencephalitis	Zhang, et al.
asparagine	Blood/serum	EOS sepsis > Control	Mardegan, et al.
butane-2,3-dione	Stool	Gram neg LOS > control	Frerichs, et al.
cadaverine	Blood/serum	EOS sepsis > Control	Mardegan, et al.
cellulose	Stool	LOS sepsis < Control	Liu, et al.
citrulline	Blood/serum	EOS sepsis > Control	Mardegan, et al.
creatinine	CSF	Downregulated in sepsis with meningoencephalitis	Zhang, et al.
cystine	Blood/serum	EOS sepsis > Control	Mardegan, et al.
cytidine 5′-diphosphocholine	Blood/serum	LOS sepsis > Control	Wang, et al.
ethyl acetate	Stool	Gram-neg LOS > control	Frerichs, et al.
ethyl 2-(methylamino)acetate	Stool	Gram-neg LOS > control	Frerichs, et al.
ethyl 2-hydroxypropanoate	Stool	Gram-neg LOS > control	Frerichs, et al.
gamma-Glutamyltryptophan	Stool	LOS sepsis < Control	Liu, et al.
glycine	Blood/serum	EOS sepsis > Control	Mardegan, et al.
	Stool	LOS sepsis < Control	Liu, et al.
glycocholic acid	CSF	Upregulated in sepsis with meningoencephalitis	Zhang, et al.
heptanal	Stool	CoNS LOS < Control	Frerichs, et al.
kynurenic acid	Blood/serum	EOS sepsis > Control	Mardegan, et al.
kynurenine	Blood/serum	EOS sepsis > Control	Mardegan, et al.
lysine	Blood/serum	EOS sepsis > Control	Mardegan, et al.
methionine	Blood/serum	EOS sepsis > Control	Mardegan, et al.
N-Methydopamine	Stool	LOS sepsis < Control	Liu, et al.
N-Ribosylnicotinamide	Stool	LOS sepsis < Control	Liu, et al.
N1-AcetylSPD	Blood/serum	EOS sepsis > Control	Mardegan, et al.
ornithine	Blood/serum	EOS sepsis > Control	Mardegan, et al.
phenylalanine	Blood/serum	EOS sepsis > Control	Mardegan, et al.
phenylglyoxylic acid	CSF	Upregulated in sepsis with meningoencephalitis	Zhang, et al.
phosphatidic acid	Blood/serum	LOS sepsis > Control	Wang, et al.
phosphatidyl ethanolamine	Blood/serum	LOS sepsis > Control	Wang, et al.
phosphorylcholine	Blood/serum	LOS sepsis < Control	Wang, et al.
proline	Blood/serum	EOS sepsis > Control	Mardegan, et al.
	CSF	Upregulated in sepsis with meningoencephalitis	Zhang, et al.
prolylhydroxyproline	Blood/serum	LOS sepsis < Control	Wang, et al.
prop-1-ene	Stool	Gram-neg LOS < control	Frerichs, et al.
sarcosine	Blood/serum	EOS sepsis > Control	Mardegan, et al.
serine	Blood/serum	EOS sepsis > Control	Mardegan, et al.
serotonin	Blood/serum	EOS sepsis > Control	Mardegan, et al.
spermidine	Blood/serum	EOS sepsis > Control	Mardegan, et al.
sphingomyelin	Blood/serum	LOS sepsis < Control	Wang, et al.
taurine	CSF	Upregulated in sepsis with meningoencephalitis	Zhang, et al.
tryptamine	Blood/serum	EOS sepsis > Control	Mardegan, et al.
tyramine	Blood/serum	EOS sepsis > Control	Mardegan, et al.
tyrosine	Blood/serum	EOS sepsis > Control	Mardegan, et al.
Unidentified metabolite 1 (m/z_RT 594.378_9.2)	Blood/serum	LOS sepsis > Control	Thomaidou, et al.
Unidentified metabolite 2 (m/z_RT 638.939_9.0)	Blood/serum	LOS sepsis > Control	Thomaidou, et al.
valine	Blood/serum	EOS sepsis > Control	Mardegan, et al.
xanthurenic acid	Blood/serum	EOS sepsis > Control	Mardegan, et al.

Disruptions in amino acid metabolism were also consistently reported across studies. Mardegan et al. (54) and Wang et al. (53) both highlighted overrepresentation of the aminoacyl-tRNA biosynthesis pathway in neonates with sepsis. Mardegan et al. (54) specifically noted elevated taurine metabolism in EOS, a finding that was also mirrored by Zhang et al. (55), who observed increased taurine in the CSF of neonates with sepsis with meningoencephalitis (ME). Both Mardegan et al. (54) and Liu et al. (56) also reported increased glutathione metabolism in neonates with EOS and LOS.

Increased metabolism of aromatic compounds, long considered a biochemical signature of sepsis, was another consistent theme. CSF analysis of neonates with ME performed by Zhang et al. (55) revealed elevated levels of phenylglyoxylic acid, a metabolite derived from phenylacetic acid, which itself is a byproduct of phenylalanine catabolism (Table 10). Liu et al. (56) similarly reported increased glyoxylic acid metabolism in neonates with LOS. Mardegan et al. (54) identified enhanced phenylalanine metabolism and also reported increased catabolism of other aromatic amino acids such as tyrosine and tryptophan. Liu et al. (56) further identified an increase in gamma-glutamyl tryptophan, underscoring the systemic alteration of aromatic amino acid pathways in neonates with sepsis.

Lastly, changes in bile acid metabolism were noted in both Liu et al. and Zhang et al. Liu et al. (56) identified primary bile acid synthesis as one of thirteen significantly involved pathways in LOS, while Zhang et al. (55) reported elevated levels of glycocholic acid, a primary bile acid, in the CSF of neonates with sepsis and ME compared to those without neurological involvement (Table 10).

## 4 Discussion

### 4.1 Microbiome

Recent advancements in microbiome sequencing have significantly enhanced our understanding of the gut microbial landscape in neonates and its potential role in the pathogenesis of sepsis. The emerging consensus across multiple studies points to a consistent reduction in alpha diversity among neonates with sepsis, suggesting that early microbial imbalance may contribute to heightened vulnerability to infection. This trend was particularly evident in infants born preterm, where microbial diversity was markedly reduced compared their counterparts born at term. The predominance of *Proteobacteria* in healthy neonates born preterm contrasted with the *Firmicutes* and *Actinobacteria* dominance seen in infants born at term (29), suggesting that gestational age plays a critical role in shaping early microbial communities. Moreover, the enrichment of Bacilli class organisms, especially Staphylococcus species such as *S. epidermidis* and *S. aureus*, in both gut and nasal microbiomes prior to sepsis onset (31, 32), underscores their potential involvement in disease progression.

Analysis of alpha diversity also strengthens the hypothesis of endogenous microbial translocation as a plausible route of infection, as dominant Staphylococcus populations were found to precede the onset of bacteremia, observed in both fecal and nasal samples. Strain-specific PCR supports this notion by confirming the presence of *S. epidermidis*, *Klebsiella pneumoniae*, and *Escherichia coli* in the gut prior to bloodstream infection (29). These findings may substantiate the concept of bacterial translocation from the gastrointestinal tract, demonstrated with molecular precision.

Cross-comparison with metabolomic findings reinforce the idea of translocation from the neonatal gut as a source of bloodstream infection. Graspeuntner et al. (31) detected in neonates with sepsis elevated fecal levels of ethanol and formic acid, likely fermentation byproducts of Bacilli class organisms, which commonly reside in the gut.

Moving beyond analysis of gut microbiota composition, interpretation of use of antibiotics suggests a deleterious effect on the growth of beneficial organisms. Several studies highlighted the suppressive effects of perinatal and early life antibiotic administration on beneficial genera such as *Lactobacillus* and *Bifidobacterium* (29, 33–35). Notably, the absence or severe depletion of *Bifidobacterium* in neonates born preterm with sepsis (33), as well as the reduced abundance of *Lactobacillus* in neonates exposed to antibiotics or born to Group B Streptococcus-positive mothers (36), underscores the fragility of microbial populations in this vulnerable group. The inverse relationship between probiotic supplementation and these depletions further highlights the potential utility of microbial restoration strategies.

Despite the documented shifts in microbial profiles and diversity, the relationship between antibiotic exposure and the emergence of causative pathogens remains complex. While certain studies linked recent antibiotic use to subsequent bloodstream infections with *Enterobacteriaceae* (38), others did not observe clear changes in pathogen abundance before or after sepsis onset (37). Furthermore, the persistence of clinical stability following antibiotic discontinuation in several cases raises important questions regarding microbial resilience and the threshold at which dysbiosis translates into systemic infection.

While attention has recently turned to novel therapeutic avenues such as lantibiotics (57, 58), bacterially derived antimicrobial peptides with broad-spectrum efficacy, their clinical application in neonatal sepsis remains largely theoretical. Though promising in preclinical models, their role in modulating early-life microbiota or preventing sepsis has not yet been explored in neonatal cohorts.

Limitations of microbiome studies include collection of samples in relation to the timeline of disease progression. All studies noted collection of samples before and after either bloodstream infection or sepsis onset with the exception of one (31), in which samples were only collected after the detection of bloodstream infection. Timing of sample collection impacts the ability to associate dysbiotic changes with sepsis onset. Moreover, some studies chose to define sepsis as presence of clinical features along with a positive blood culture, while some studies defined cases solely by the presence of a positive bloodstream infection. Lastly, the use of maternal factors in certain studies as an independent variable of study makes comparison of results to those in which neonates are the primary subject difficult. Nonetheless, these studies underscore the complex interplay between microbial colonization patterns, antibiotic exposure, and the risk of neonatal sepsis. While the precise mechanistic links remain to be fully elucidated, these findings support continued exploration of microbiome-targeted interventions, such as probiotics and potentially lantibiotics, as part of a comprehensive strategy to mitigate sepsis risk in neonates, particularly those born preterm.

### 4.2 Genomics

Genomic research has recently expanded in scope, encompassing both pathogen and host related investigations. While still in its early stages compared to other omics approaches, the application of WGS to neonatal sepsis is beginning to uncover valuable insights into microbial virulence, host susceptibility, and antiseptic resistance patterns. The limited number of studies meeting inclusion criteria in this review reflects the early onset nature of this field but also emphasizes its growing relevance.

Host-focused genomic analysis, as conducted by Ciesielski et al. (41), reveals compelling evidence of sex-specific genetic susceptibility to LOS. The identification of distinct SNPs in male and female neonates, without overlap between the two, suggests potential differences in immune regulation or response pathways that may be inherently linked to sex chromosomes or sex dependent gene expression. Notably, several of the variants mapped to components of the NOTCH signaling pathway (41), which has known roles in T-cell development and apoptotic processes, both of which are critical to immune function. Additionally, SNPs in genes encoding IL-10 and TNF-α (41), two major cytokines involved in the neonatal inflammatory response, underscore the potential for inherited variation in immune signaling to shape infection outcomes. These findings provide a genomic framework for understanding individual variability in sepsis risk and open the door for future studies examining personalized approaches to sepsis prevention or treatment based on genetic profiling.

On the microbial side, the work by Lipworth et al. (39) adds an important dimension to pathogen genomics, specifically in relation to vaccine development. Their finding that a large subset of *E. coli* isolates from neonates wtih sepsis harbored O-antigen serotypes included in the ExPEC-4V vaccine formulation suggests that maternal immunization strategies, already being trialed in adults (59), may be repurposed to protect neonates against bloodstream infections. Continued clinical testing of the ExPEC-4V and other O-antigen vaccines in adult populations may yield results that could eventually be extended to neonates. If successful, this could provide a practical and highly targeted tool to reduce the burden of *E. coli*-mediated sepsis in this vulnerable group.

Though the focus of the study by Sethi et al. (40) differs, its findings also contribute to the broader goal of neonatal sepsis prevention. By identifying elevated chlorhexidine minimum inhibitory concentrations in UK isolates (40), where antiseptic use is more prevalent, the study highlights the potential for antiseptics to exert selective pressure on microbial populations. While no specific resistance genes were definitively associated with chlorhexidine or octenidine tolerance (40), the results imply that resistance may be developing through mechanisms not yet understood. This underscores the need for more prudent selection of antiseptics in neonatal intensive care settings to prevent the emergence of resistant strains. Preserving the effectiveness of these compounds could be critical to maintaining infection control measures and improving outcomes in neonates with sepsis.

Together, the findings from Lipworth et al. (39) and Sethi et al. (40) offer two complementary angles for advancing sepsis prevention: immunization to reduce infection risk and stewardship of antiseptic use to limit resistance. Both studies demonstrate how genomic tools can inform strategies that go beyond treatment to encompass proactive measures in infection control.

While the primary focus of this review was on biomarker and susceptibility research, it is worth noting the wealth of studies employing WGS for outbreak tracking in NICUs (Table 4). Though excluded from the core analysis due to their epidemiological nature, these studies reinforce the practical utility of genomics in infection control and could serve as a foundation for future biomarker discovery through strain specific virulence factor analysis.

Comparison of the genomic studies is limited by the timing of sample collection, as all studies did not collect samples until the onset of bloodstream infection or sepsis diagnosis. Whereas one study solely defined sepsis criteria as presence of bloodstream infection, another included abnormal clinical and laboratory findings. The third study was of microbial focus and included all staphylococcal isolates admitted to two NICUs over a 10-week period. The heterogeneity of sepsis definition also limits the ability to draw parallels between findings of the studies.

Altogether, these genomic studies underscore the multifaceted utility of sequencing technologies in neonatal sepsis research. Whether through identifying host risk alleles, monitoring the impact of antimicrobial interventions, or informing future vaccine strategies, genomics is poised to become an integral component of precision-based neonatal care. As research in this area progresses, it may lead not only to improved understanding of disease pathophysiology but also to more effective, individualized approaches to sepsis prevention and management.

### 4.3 Transcriptomics

Transcriptomic technologies offer a dynamic snapshot of the host immune response to infection and have revealed promising gene-level insights into the pathophysiology of neonatal sepsis. The studies reviewed collectively highlight key immune pathways activated during infection, and although variability in study design limits direct comparisons, several recurring molecular signals emerge that may inform biomarker development and therapeutic interventions.

Among these signals, *CD3G* emerged as the most consistently reported gene across studies (43, 44). *CD3G* encodes the CD3-gamma subunit of the T-cell receptor complex, an essential component for T-cell antigen recognition and activation. Its downregulation in neonates with EOS relative to healthy controls (44) and in septic shock compared to bacteremia (43) suggests a broader role for T-cell dysfunction in sepsis progression. Moreover, these observations may represent a continuum of *CD3G* modulation along the clinical spectrum from healthy neonate to neonate with sepsis to septic shock. The observed initial CD3G suppression in EOS could contribute to a transient immunodeficient state, facilitating bacterial translocation into the bloodstream. As the neonate progresses through infection, upregulation of CD3G expression in septic shock may reflect a dysregulated immune response or compensatory overactivation, contributing to cytokine storm and tissue damage. Future studies examining CD3G expression longitudinally across sepsis stages, and specifically within neonates with bacteremia who do or do not progress to shock, may help establish its value as a predictive biomarker of disease severity.

Despite limited overlap in specific genes across studies, multiple investigations reported genes involved in similar immunologic pathways, reinforcing shared biological mechanisms of neonatal sepsis. For example, *CXCL10*, a chemokine that recruits T cells, natural killer cells, and monocytes, was found upregulated in neonates with bacteremia but not in those with septic shock (43). This suggests that more robust leukocyte recruitment may be protective, consistent with the improved outcomes observed in infants with bacteremia compared to those experiencing systemic decompensation. Similarly, *CSK*, identified by Cernada et al. (45) as upregulated in gram-positive sepsis, promotes immune cell migration and may exert anti-apoptotic effects. Together, these findings suggest a common immunoprotective role for leukocyte migration, particularly in less severe forms of sepsis or gram-positive infections. This aligns with clinical observations of better prognosis in bacteremia and gram-positive infections (60, 61) and suggests that modulation of immune cell trafficking could be an avenue for future therapeutic intervention.

In addition to specific immune-related genes and shared immune pathway involvement, inflammatory pathway activation also emerged as a recurrent theme. Huang et al. (43) reported upregulation of *MM8* in neonates with septic shock, a gene known to activate NF-*κ*B, a transcription factor central to inflammatory cytokine production. Complementarily, Ng et al. (23) observed increased expression of NF-*κ*B1A, a downstream target of MM8, in neonates with LOS. This linkage between studies points to an MM8–NF-*κ*B signaling axis as a potential driver of the exaggerated inflammatory response in severe sepsis. Further exploration of this pathway could clarify the mechanisms underpinning sepsis-associated organ dysfunction and identify novel targets for immunomodulation.

A fourth major theme involved IFN-1 signaling, highlighted in both Qi et al. and Ng et al. Qi et al. (62) found upregulation of IFN-1 pathways, including “cell response to type I interferon” and “negative regulation of viral genome replication”, in nonsurvivors of neonatal sepsis. Ng et al. (23) reported increased expression of genes that induce IFN-1 production in neonates with LOS, but this was not matched by elevated protein levels of IFN-α or IFN-β, suggesting post-transcriptional regulation or feedback inhibition. When examined together, these findings illustrate different stages of the IFN-1 signaling cascade, with Ng et al. (23) capturing the early transcriptional priming, and Qi et al. (62) identifying the functional consequences and associated mortality. This cross-study synthesis underscores the need for multi-omic validation to confirm findings at the protein and functional levels.

Beyond mechanistic insights, transcriptomics holds clinical promise for improving the diagnostic accuracy and risk stratification of neonatal sepsis. Several studies, including Huang et al. (43), proposed genes with potential predictive value for septic shock, though validation in larger, prospective cohorts is necessary. Combining transcriptomic biomarkers with proteomic, genomic, or metabolomic data may improve prediction models that distinguish neonates with sepsis at risk of deterioration from those likely to recover. Particularly, modular gene signatures, which capture specific immune responses such as T-cell suppression, leukocyte recruitment, or cytokine signaling, may be more effective than single gene markers in capturing the heterogeneity of neonatal sepsis.

A strength offered by the transcriptomic studies is the analysis of neonates through the stages of sepsis, from bloodstream infection to sepsis to shock. Such longitudinal analysis allows for association of transcriptomic disruption with specific points along the timeline of disease. However, two studies only collected samples after the onset of bloodstream infection or sepsis diagnosis, and three used data from public datasets, in which individual data was compiled from multiple studies, each with its own definition of sepsis.

In summary, transcriptomic profiling has uncovered key immune pathways involved in neonatal sepsis, including T-cell receptor signaling (*CD3G*), leukocyte chemotaxis (*CXCL10, CSK*), inflammatory activation (*MM8*, NF-κB1A), and IFN-1-mediated immune modulation. While further research is needed to translate these insights into clinical tools, the convergence of findings across independent studies strengthens the case for transcriptomics as a critical platform in the development of personalized sepsis diagnostics and interventions in the neonatal population.

### 4.4 Mirnomic

Although still an emerging field, miRNomic analysis offers valuable insights into the post-transcriptional regulation of immune responses during neonatal sepsis. The reviewed studies highlight the role of differentially expressed miRNAs in modulating key components of the innate immune system in neonates with sepsis. Despite differing in study populations and sepsis onset (EOS vs. LOS), both investigations converge on the importance of miRNAs in regulating TLR signaling pathways, which are central to neonatal immune defense against bacterial infections.

While Janec et al. (47) reported upregulation of miRNAs known to suppress elements of the TLR4 signaling cascade in neonates with EOS (63, 64), Serna et al. (49) observed downregulation of multiple immune-regulatory miRNAs in infants with VLBW and LOS. These included miR-23, miR-17, and miR-15a. miR-23 influences innate immunity via regulation of metalloproteinase 10, a protein involved in tissue remodeling and inflammatory responses (65). miR-17 has broader immunomodulatory functions, including impacts on T-cell proliferation and function (66), while miR-15a is known to suppress both TLR4 and interleukin-1 receptor-associated kinase 1, another key component of TLR signaling (67). The under expression of these miRNAs in LOS may lead to unchecked inflammatory signaling and exaggerated immune responses, which are commonly observed in neonates with sepsis. The contrasting expression patterns observed across EOS and LOS may indicate differences in host immune maturity, pathogen exposure, or temporal dynamics of sepsis development. It is also possible that miRNA profiles reflect divergent immune strategies, suppressing inflammation in EOS to prevent collateral tissue damage, vs. failing to adequately restrain inflammation in LOS, leading to immune dysregulation.

Despite contrasting findings, both studies highlight the regulatory influence of miRNAs on TLR4 signaling (40, 47), underscoring a shared molecular axis in sepsis pathophysiology. Given that overactivation and under-activation of TLR pathways can be detrimental in sepsis (68, 69), maintaining an optimal level of immune activation appears critical. Synthesis and comparison of the results from these two studies highlights the therapeutic potential of miRNAs as modulators of immune balance. For instance, restoring the expression of downregulated miRNAs in LOS or selectively inhibiting overexpressed miRNAs in EOS could offer a targeted approach to modulate immune responses without broadly suppressing immunity.

Conclusions drawn from the miRNA studies are strengthened by the shared use of blood samples as well as shared definition of sepsis by a positive blood culture. However, the small number of studies calls for more research to be done utilizing this molecular method. Fortunately, miRNAs show potential for future research, as they are stable in circulation and amenable to non-invasive sampling, making them attractive candidates for biomarker development. Their differential expression in neonates with and without sepsis, as demonstrated in both studies (40, 47), suggests that miRNA panels could support early diagnosis or stratification of sepsis severity. Future studies should validate these findings in larger cohorts and explore whether specific miRNA signatures are predictive of clinical outcomes such as progression to septic shock, need for intensive care interventions, or mortality.

### 4.5 Epigenomics

Genome-wide methylation analysis performed in epigenomic studies offers insights into changes in DNA expression that may provide an upstream explanation of changes in neonates with sepsis demonstrated by other omic analyses. The hypermethylation of the *CD3G* gene promoter in neonates with sepsis (48) aligns with transcriptomic analyses showing decreased expression of *CD3G* in neonates with EOS compared to neonates without sepsis (44) and in neonates with septic shock compared to neonates with bacteremia (43). Epigenomic analysis offers a potential explanation for the differential expression observed in transcriptomic analysis.

Results of epigenomic analysis may also underscore findings from other omic methods. When taken together, the hypomethylation of the *IL10* promoter (48) in neonates with sepsis and the SNPs identified in genes encoding IL-10 in neonates with LOS compared to controls (41) suggest a multifactorial explanation of aberrant immune function that may place certain neonates at higher susceptibility of developing sepsis. Additionally, hypomethylation of the *MIR886* promoter in LOS (48) may lead to increased expression of microRNA miR-886-5p seen in miRNomic analysis. miR-886-5p has been shown to downregulate *BAX,* inhibiting apoptosis (70), and the increase in expression may be another mechanism of aberrant immune function increasing susceptibility*.* Thus, epigenomic analysis may provide another pathway to study the disruption of apoptotic regulation associated with changes in NOTCH signaling observed on genomic analysis of neonates with LOS (41).

Cross-comparison of epigenomics with miRNomics may also provide insight into the disrupted immune regulation present in sepsis. Lorente-Pozo et al. (48) report hypomethylation of the promoter for *MIR10A* encoding the microRNA miR-10a, which has been shown to negatively correlate with production of pro-inflammatory cytokines (71). The hypomethylation of the *MIR10A* promoter in LOS (48) may work in tandem with the decreased expression of immune regulatory miRNAs observed in miRNomic analysis of neonates with LOS (46), ultimately creating a setting of extreme inflammation in sepsis. Epigenomic studies provide a way to approach neonatal sepsis pathogenesis from a multifactorial standpoint. However, the conclusions drawn here are severely limited by the fact that only one epigenomic study met criteria for inclusion in our review. That said, comparison with findings across other omics suggest potential relationships between transcriptomic, genomic, and miRNomic analyses. Future studies may aim to further elucidate these relationships.

### 4.6 Proteomics

Proteomic profiling has emerged as a powerful approach to uncover the molecular alterations associated with neonatal sepsis. By directly quantifying protein expression, proteomics can offer insight into both systemic inflammation and organ specific responses that may not be captured by genomic or transcriptomic methods.

Chatziioannou et al. (49) examined serum proteomes in neonates with LOS and identified three candidate biomarkers: APOA4, CETP, and CRP. These findings reflect key aspects of the host's inflammatory and metabolic responses to infection. The downregulation of APOA4 aligns with patterns previously observed in adult sepsis, where decreases in apolipoproteins are thought to reflect inflammation induced disruption of lipid transport and metabolism (49). Similarly, reduced CETP levels, known to correlate with worse outcomes in adult sepsis (49), suggest an impaired ability to manage lipid trafficking during the systemic inflammatory response. CRP, a well-established marker of inflammation, supports the validity of this proteomic panel and highlights the relevance of combining novel markers with conventional ones to enhance diagnostic accuracy.

In contrast, Jiang et al. (50) focused on CSF to investigate proteomic changes associated with neuroinflammation in neonates with sepsis. Their study revealed significant downregulation of proteins critical for maintaining neuronal structure and extracellular membrane (ECM) integrity (50). Among these were MMP-2, which regulates ECM remodeling, and cystatin C, a protective protease inhibitor involved in CNS homeostasis (50). The additional decreases in structural ECM proteins such as brevican, nidogen-1 and −2, and fibronectin suggest that neuroinflammatory damage in neonatal sepsis may involve degradation of the ECM (50), potentially contributing to long-term neurological complications.

Together, these studies emphasize the multifaceted nature of neonatal sepsis, wherein systemic inflammation, metabolic dysregulation, and neuroinflammation can all be reflected in proteomic changes. Importantly, the biological fluid selected for analysis, serum vs. CSF, yields insights into distinct compartments of disease activity. This supports the notion that a multi-compartmental approach to proteomic sampling may be valuable in fully characterizing neonatal sepsis, especially in cases with suspected or confirmed CNS involvement.

The identification of APOA4 and CETP as potential serum biomarkers for LOS (49) represents a promising step toward developing more precise diagnostic tools. Given that many laboratory tests evaluating for sepsis lack specificity or only become positive after clinical deterioration (72, 73), early detection using proteomic signatures may improve timely intervention and outcomes. Additionally, the downregulation of ECM related proteins in CSF points to possible mechanisms underlying sepsis-associated encephalopathy and may offer targets for therapeutic intervention or prognostic stratification.

Despite their contributions, both studies are limited by relatively small sample sizes and require validation in larger, diverse cohorts. Additionally, one study defined sepsis as the presents of two or more abnormal laboratory findings from a pre-determined list, while the other diagnosed sepsis by clinical and laboratory findings. Furthermore, proteomic data must ultimately be integrated with other omics approaches to obtain a comprehensive understanding of disease mechanisms.

### 4.7 Metabolomics

Metabolomic profiling has emerged as a tool for uncovering the biochemical underpinnings of neonatal sepsis, offering insight into the interplay between infection, inflammation, and systemic physiological responses. The studies reviewed consistently demonstrate that neonatal sepsis, whether early onset, late onset, or complicated by meningoencephalitis, is characterized by multifaceted disruptions in metabolic pathways. Key findings included alterations in lipid and amino acid metabolism, changes in aromatic compound catabolism, and disruptions in bile acid pathways, which collectively provide diagnostic and mechanistic insight into the disease.

Lipid metabolism was the most commonly affected pathway across studies (52, 53). Reductions in PC, lysoPC, and sphingomyelin were reported in neonates with LOS. These lipids play critical roles as structural components of cellular membranes but also as enzyme activators and anti-inflammatory mediators (74, 75). The decrease in lysoPC and PC reported by Thomaidou et al. (52) aligns with Wang et al.'s (53) observation of reduced sphingomyelin, another essential membrane lipid. Notably, sphingomyelin was highlighted as a strong predictor of sepsis mortality (53), further supporting its clinical relevance. While Wang et al. (53) also reported an increase in total PC levels and its precursor CDP-CHO, this finding may reflect differential use of CDP-CHO in other biosynthetic or stress response pathways during LOS. Together, these results reinforce the notion that disrupted lipid metabolism is a defining feature of neonatal sepsis and may provide targets for early detection and risk stratification.

Amino acid metabolism was similarly affected, particularly those pathways related to stress response and immune modulation. Taurine metabolism was notably upregulated in both blood and CSF, with Zhang et al. (55) and Mardegan et al. (54) reporting elevated levels in neonates with sepsis and those with ME. Taurine is a well-known antioxidant with protective effects on mitochondria, suggesting that its upregulation may be a response to sepsis induced oxidative stress. However, the overproduction of antioxidants, as seen in neonates with ME (55), raises the possibility that excessive antioxidant activity may also contribute to pathological processes such as neuroinflammation. This interpretation is supported by the concurrent upregulation of glutathione metabolism in both EOS and LOS (54, 56), further indicating a system wide oxidative stress response in sepsis, regardless of timing.

Another theme across studies was the alteration of aromatic amino acid metabolism. Phenylglyoxylic acid, a metabolite of phenylacetic acid and a downstream product of phenylalanine catabolism, was found to be elevated in neonates with ME (55). Phenylalanine has been shown to stimulate pro-inflammatory cytokine release (76, 77), supporting a link between elevated phenylglyoxylic acid levels and heightened inflammatory responses in sepsis. Liu et al. (56) similarly reported increased glyoxylic acid metabolism, and Mardegan et al. (54) identified enhanced breakdown of aromatic amino acids including phenylalanine, tyrosine, and tryptophan. Interestingly, not all derivatives of these pathways are purely proinflammatory. Gamma-glutamyl tryptophan, for instance, has been described as possessing anti-inflammatory and immune supportive properties (56), highlighting the dual and complex roles of these metabolites. These observations suggest that neonatal sepsis involves an interplay between pro inflammatory and anti-inflammatory mechanisms, rather than a unidirectional immune activation.

Disruptions in bile acid metabolism were also noted, providing a possible link between gut health and systemic inflammatory responses. Elevated glycocholic acid levels in the CSF of neonates with ME (55) and upregulation of primary bile acid synthesis pathways in LOS (56) point to potential gut-liver-brain axis involvement in sepsis. Bile acids are known to modulate inflammation and regulate the gut microbiota, and their dysregulation may contribute to the leaky gut phenomenon proposed by Graspeuntner et al. (31). However, it is important to note that samples in these studies were collected after clinical suspicion or onset of sepsis, limiting the ability to determine whether bile acid changes are a cause or consequence of infection. Nevertheless, given that bile acids facilitate lipid absorption, disruptions in bile acid metabolism may lie upstream of the lipid metabolic changes described earlier, suggesting that multiple metabolomic findings may be linked through interconnected pathways.

Many of the reported metabolic changes were consistent across both EOS and LOS, indicating shared pathophysiologic mechanisms and limiting the utility of some biomarkers in distinguishing onset stage. However, several metabolites, particularly those measured in CSF, showed potential to differentiate between sepsis with and without neurologic complications such as ME. For example, increased taurine and phenylglyoxylic acid in ME cases (55) suggest that metabolic profiling may help predict complications of sepsis and guide neuroprotective interventions.

Metabolomics provides compelling evidence that neonatal sepsis is characterized by widespread alterations in lipid, amino acid, and bile acid metabolism. These findings support a complex, multi system model of disease involving proinflammatory, anti-inflammatory, oxidative stress, and potential organ specific vulnerabilities.

All metabolomic studies included a positive blood culture as a requirement for sepsis diagnosis, and none relied solely on a positive culture to make the diagnosis. However, only two studies collected samples before suspicion or confirmed diagnosis of sepsis; the rest collected the first sample upon diagnosis of sepsis. Future studies should explore longitudinal changes in metabolite profiles, incorporate pre-symptomatic sampling when feasible, and integrate findings with other omics data to construct a holistic view of sepsis pathogenesis. Ultimately, these efforts may lead to improved diagnostic tools and targeted therapies that reduce sepsis related morbidity and mortality in neonates.

## 5 Conclusion

The expanding field of molecular research has advanced our understanding of neonatal sepsis to offer novel insights into its diagnosis, pathogenesis, and potential avenues for prevention and treatment. Each branch of molecular science contributes uniquely to the broader picture. Transcriptomic studies enhance diagnostic precision and risk stratification by identifying gene expression signatures associated with sepsis severity and outcomes, such as shock. Microbiome analyses highlight the role of gut dysbiosis in sepsis susceptibility, shedding light on how early microbial colonization, antibiotic exposure, and probiotic use may influence infection risk. Metabolomic studies continue to uncover promising biomarkers for early diagnosis and detection of sepsis-related complications, while proteomic investigations corroborate these findings by identifying altered protein expression patterns with prognostic value. Genomic studies offer potential for personalized prevention strategies, particularly in identifying inherited vulnerabilities, and often reinforce key findings from transcriptomic and metabolomic work. Meanwhile, miRNomic research provides mechanistic insight into post-transcriptional regulation of immune responses, further refining our understanding of neonatal host-pathogen interactions.

Looking ahead, future molecular research methods should focus on distinguishing mechanisms leading to EOS from mechanisms contributing to LOS, an area where current biomarkers lack sufficient specificity. Additionally, greater attention is needed to delineate the cascade of inflammatory processes that unfold during disease progression and to identify biomarkers predictive of long-term complications, such as neuroinflammation. Integration of multi-omics approaches holds particular promise for constructing a comprehensive systems-level model of neonatal sepsis. While significant progress has been made, molecular science still has much to offer in transforming neonatal sepsis care, from more accurate and timely diagnosis to targeted interventions and personalized therapies that improve outcomes for this vulnerable population.

## Data Availability

The original contributions presented in the study are included in the article/Supplementary Material, further inquiries can be directed to the corresponding author.
